# Analyses of soil microbial community compositions and functional genes reveal potential consequences of natural forest succession

**DOI:** 10.1038/srep10007

**Published:** 2015-05-06

**Authors:** Jing Cong, Yunfeng Yang, Xueduan Liu, Hui Lu, Xiao Liu, Jizhong Zhou, Diqiang Li, Huaqun Yin, Junjun Ding, Yuguang Zhang

**Affiliations:** 1 Institute of Forestry Ecology, Environment and Protection, and the Key Laboratory of Forest Ecology and Environment of State Forestry Administration, the Chinese Academy of Forestry, Beijing 100091, China; 2 School of Minerals Processing and Bioengineering, Central South University, Changsha 410083, China; 3 State Key Joint Laboratory of Environment Simulation and Pollution Control, School of Environment, Tsinghua University, Beijing 100084, China; 4 Institute for Environmental Genomics and Department of Botany and Microbiology, the University of Oklahoma, Norman OK 73019.

## Abstract

The succession of microbial community structure and function is a central ecological topic, as microbes drive the Earth’s biogeochemical cycles. To elucidate the response and mechanistic underpinnings of soil microbial community structure and metabolic potential relevant to natural forest succession, we compared soil microbial communities from three adjacent natural forests: a coniferous forest (CF), a mixed broadleaf forest (MBF) and a deciduous broadleaf forest (DBF) on Shennongjia Mountain in central China. In contrary to plant communities, the microbial taxonomic diversity of the DBF was significantly (*P* < 0.05) higher than those of CF and MBF, rendering their microbial community compositions markedly different. Consistently, microbial functional diversity was also highest in the DBF. Furthermore, a network analysis of microbial carbon and nitrogen cycling genes showed the network for the DBF samples was relatively large and tight, revealing strong couplings between microbes. Soil temperature, reflective of climate regimes, was important in shaping microbial communities at both taxonomic and functional gene levels. As a first glimpse of both the taxonomic and functional compositions of soil microbial communities, our results suggest that microbial community structure and function potentials will be altered by future environmental changes, which have implications for forest succession.

In ecological theory, succession indicates the somewhat orderly and predictable species changes in space and time following the colonization of a new environment^1^, and it has been one of the central ecological topics for over a century^2^. To date, the vast majority of research on succession has focused on plant communities, which is defined as the succession of species composition and vegetation characteristics^3^. Plant productivity, biomass, nutrient availability, landscape-level heterogeneity, light availability, soil structure, species traits, and inter-species interaction are important for plant community succession resulting from environmental changes^4^,^5^,^6^. For many studies, the use of space-for-time substitution (chronosequence) succession, despite recent criticisms^7^, has offered a unique opportunity to examine soil macro-community development in terms of biodiversity^8^, nutrient cycling^9^, natural and anthropogenic disturbances^10^ and land-use changes^11^ across multiple time scales.

Thus far, few studies have assessed the successional patterns of soil microbial communities along environmental chronosequences. Microbial communities are able to respond more rapidly than plant communities to environmental changes, which in turn affect ecosystem processes, such as carbon and nitrogen cycling, because of the vastness of microbial biomass and diversity^12^,^13^. Therefore, integrating microbial communities into the study of ecological succession would provide much-needed knowledge^2^. In recent years, several studies of soil microbial succession have been reported^13^,^14^. One study identified a successional trend in a bacterial community at the phylum level during forest ecosystem restoration based on a 16S rRNA gene microarray analysis^15^. Another study assessed microbial community structure by analyzing phylogenetic microarrays across chronosequences of secondary succession of chalk grasslands^14^. A third study evaluated microbial community succession by targeting 16S rRNA genes, via 454 pyrosequencing, in the soil microcosms of a desert oasis^13^. However, these studies yielded contradictory results regarding community-level diversity during succession. In addition, all of the studies were based on 16S rRNA gene analysis; thus, it remains unclear how microbial metabolic potentials are affected during succession^13^,^14^.

It is worth mentioning that microbial succession involves species interactions, which form complicated networks through various types of associations, such as predation, competition and mutualisms^16^. However, defining the network structure in a microbial community is challenging because of its high level of diversity, although this has been alleviated by rapid advances in high-throughput technologies that have been used to analyse microbial community structures^17^,^18^. Recently, conceptual frameworks for examining the network interactions of microbial communities have been generated and used to study microbial ecology^19^,^20^. Random matrix theory-based algorithms have been used to analyse soil microbial communities from macro datasets. The resulting ecological association networks have been shown to be sensitive, reliable, and robust^21^.

Here we report an integrated study with MiSeq sequencing to target 16S rRNA genes; a functional gene array to target approximately 142,000 genes involved in biogeochemical carbon, nitrogen, phosphorus and sulfur cycles (GeoChip 4.0); and network analyses to examine soil microbial communities. The study site is located on Shennongjia Mountain, a border region between China’s subtropical and temperate climate zones^22^. Because of its unique geographic location and complex topography, this area is home to variety of vegetation types and is considered to be one of the most biodiverse areas in China^23^. We selected three typical adjacent forest types, namely, a coniferous forest (CF), a mixed broadleaf forest (MBF) and a deciduous broadleaf forest (DBF), which are major forest types of the world forest zones, whose succession is influenced by environmental changes^24^. The aims of our study were to determine: (i) the soil microbial community structure, functional potentials and network interactions in three adjacent natural forest types; and (ii) the major environmental factors in shaping soil microbial community structure and functional potentials. To our knowledge, this is the first study to analyse in-depth the taxonomic and functional compositions of soil microbial communities in adjacent natural forest types, which has major implications for predicting the consequences of potential forest succession.

## Results

### Plant diversity and soil geochemical properties

The plant communities and the soil geochemical properties of the three natural forest types were analysed (Supplementary Table S1). The alpha-diversity of plant communities, as measured by the Shannon and Simpson indices, was significantly (*P* < 0.05) higher in the MBF than in the CF and DBF (Supplementary Table S1). The most dominant communities of trees and shrubs in the CF, MBF, and DBF were *Abies fargesii-Syringa reflexa*, *Acer maximowiczii-Schisandra incarnate*, and *Carpinus viminea-Viburnum erosum*, respectively (Table 1). In contrast, soil organic carbon (SOC), total nitrogen (TN), available nitrogen (AN), rapid available phosphorus (RAP), nitrate nitrogen (NN), and labile organic carbon (LOC) were significantly lower (*P* < 0.05) in the DBF than in the CF and MBF (Supplementary Table S1). Soil temperature at a depth of 10 cm (TE10) was significantly higher in the DBF (16.30 °C) than in the CF (10.83 °C) and MBF (11.74 °C), which was consistent with the annual average air temperatures (9.50 °C, 5.90 °C, and 4.00 °C in the DBF, MBF, and CF, respectively). Soil moisture was the highest in the DBF, while soil pH showed no significant differences between the three forest types. These results showed that plant diversity and soil geochemical properties distinctly differed between the three natural forest types.

### The overall bacterial taxonomic and functional gene composition from three adjacent natural forest types

**Figure 1:**
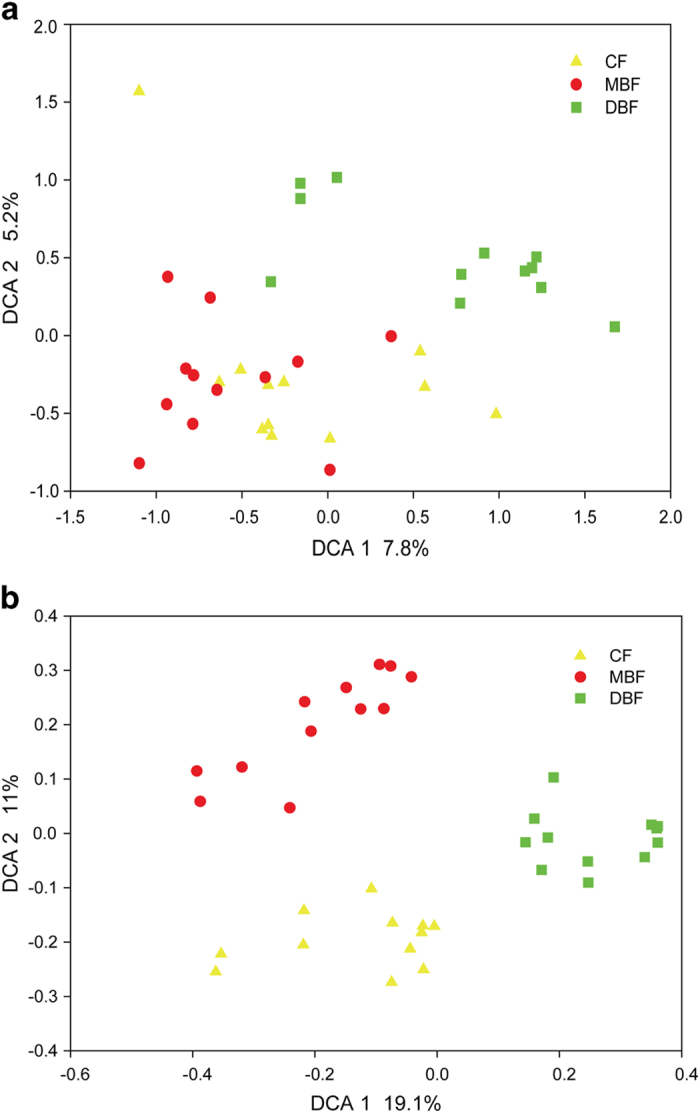
Detrended correspondence analysis (DCA) of soil microbial community based on high-throughput sequencing data (**a**) and GeoChip data (**b**). CF: coniferous forest; MBF: mixed broadleaf forest; DBF: deciduous broadleaved forest.

At the taxonomic level, 2,381,136 16S rRNA gene sequences were acquired from 36 samples from the three adjacent forest types; the number of reads ranged from 14,535 to 1,190,568 per sample. Subsequently, a total of 73,993 operational taxonomic units (OTUs) were generated at the 97% similarity level (Supplementary Table S2). There were more OTUs in the DBF (7150 ± 945) than in the CF (6542 ± 943) and MBF (6210 ± 729). Accordingly, the Shannon index was significantly higher in the DBF (7.61 ± 0.42) than in the CF (7.10 ± 0.60) and MBF (6.72 ± 0.60) (Table 2), which was in contrast to the alpha-diversity of their plant communities. *Proteobacteria*, *Acidobacteria* and *Actinobacteria* were the most abundant phylum in the soil microbial communities, followed by *Planctomycetes* and *Verrucomicrobia* (Supplementary Table S2). The abundances of many phyla, such as *Actinobacteria*, *Planctomycetes*, and *Verrucomicrobia*, were significantly higher in the DBF samples than in the CF and MBF samples (*P* < 0.05). To identify overall differences among these sites, detrended correspondence analysis (DCA) was conducted using sequencing data, which suggested that the samples were well separated from each other, with partial overlaps (Fig. 1a). These results were verified by dissimilarity tests using analysis of similarity (ANOSIM) and multiple response permutation procedure (MRPP) algorithms (Supplementary Table S3).

At the functional gene level, a total of 46,303 gene probes were detected using GeoChip 4.0. The gene richness and alpha diversity, as judged by the Shannon index, of the DBF (39,970 ± 2,250 and 10.59 ± 0.06, respectively) were significantly (*P* < 0.05) higher than those of the CF (34,221 ± 3,916 and 10.43 ± 0.12, respectively) and MBF (34,153 ± 3,551 and 10.43 ± 0.11, respectively) (Table 2). The DCA using the GeoChip data showed that the samples from three adjacent forest types were well separated from each other (Fig. 1b), which was also verified by dissimilarity tests using the ANOSIM and MRPP algorithms (Supplementary Table S3).

### Functional genes involved in carbon and nitrogen cycling

A total of 5,296 genes involved in carbon cycling genes were detected. At the gene family level, most carbon cycling genes remained unchanged (Supplementary Fig. S1). However, the differences were apparent at the probe level. Propionyl CoA carboxylase (*PCC*) was the most abundant carbon fixation gene among the three forest types. A closer examination indicated that probes targeting *PCC* were mainly derived from bacteria, except for some derived from the *Crenarchaeota* and *Euryarchaeota* phyla of archaea. CO dehydrogenase (*CODH*) was significantly (*P* < 0.05) more abundant in the CF than in the DBF, and most of the abundant *CODH* genes were derived from bacteria, except for a few derived from the *Crenarchaeota*, *Euryarchaeota*, and *Thaumarchaeota*. The most abundant *CODH* genes were derived from *Bradyrhizobium* spp., *Mycobacterium smegmatis*, *Solibacter usitatus*, *Stappia* spp., *Mesorhizobium* spp., *Silicibacter* spp., *Nocardioides* spp., *Burkholderia* spp., *Aminobacter* spp., *Ralstonia eutropha,* and uncultured bacteria, which are known to have a wide distribution^25^. The ribulose-1,5-bisphosphate carboxylase/oxygenase (*RuBisCO*) genes detected in the CF and MBF were mostly derived from bacteria, such as *Bacillus cereus* subsp., *Bradyrhizobium* spp., *Hydrogenovibrio marinus*, *Rhodobacter sphaeroides* ATCC, and *Thiobacillus denitrificans*, which are also known to have a wide distribution^26^.

Regarding carbon degradation, a variety of functional genes whose protein products are involved in degrading different carbon substrates, such as starch, pectin, hemicellulose, cellulose, chitin, and lignin, were detected, which revealed rich microbial functional potentials in the forest ecosystems (Supplementary Fig. S1). For example, *amyA*, which encodes alpha-amylase, was very abundant in all three forest types. The *amyA* probes were mainly derived from bacteria, except for some derived from fungi, such as the *Ascomycota* and *Basidiomycota*, and the archaeal phyla *Ascomycota*, and *Euryarchaeota*, which play important roles in starch degradation^27^. Regarding methane cycling, three key functional genes, *mcrA* (5-methylcytosine-specific restriction enzyme A), *mmoX* (methane monooxygenase), and *pmoA* encoding particulate methane monooxygenase, were detected by the GeoChip. Among these, the total abundance of *mcrA*, which is considered to a diagnostic indicator of methanogenesis^28^, and the methane oxidation gene *mmoX* were significantly higher in the DBF than in the CF and MBF, suggesting that perhaps the DBF had a higher functional potential for methane cycling. These genes were mostly derived from uncultured archaea and some bacterial phyla of the *Actinobacteria* and *Proteobacteria*.

**Figure 2:**
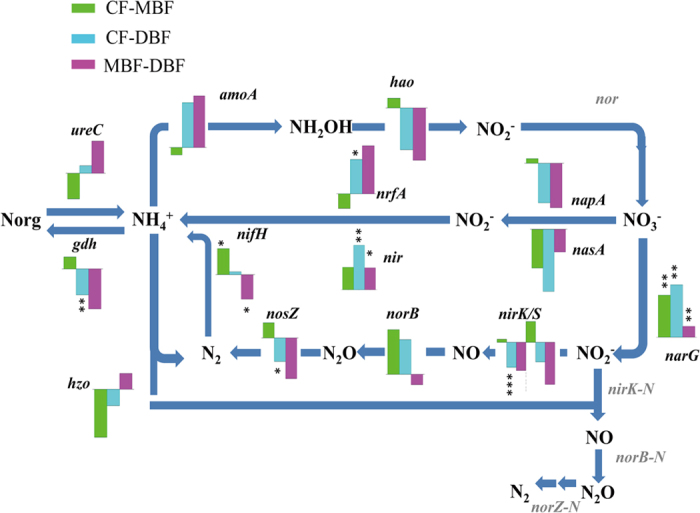
Relative changes of nitrogen cycle genes among CF, MBF and DBF. Green, blue and red colors indicate the higher/lower signal intensity of each detected gene for CF over MBF, CF over DBF and MBF over DBF, respectively. ***P < 0.001; **P < 0.01, *P < 0.05.

Regarding nitrogen cycling genes, key functional genes for assimilatory nitrogen reduction, dissimilatory nitrogen reduction, denitrification, nitrification, ammonification, anaerobic ammonium oxidation, and nitrogen fixation were detected in the three forest types (Fig. 2). The total abundances of nitrogen cycling genes did not significantly differ between the three forest types. However, the total abundances of the denitrification genes *nosZ* and *nirK* were significantly lower in the CF than in the DBF (*P* < 0.05), suggesting a shift in the microbial functional potential from nitrate biosynthesis in the CF toward ammonium biosynthesis in the DBF (Fig. 2). Consistent with this view, TN, AN, and NN concentrations were higher in the CF and MBF than in the DBF, while the ammonium concentration was higher in the DBF than in the CF and MBF (Supplementary Table S1). The most abundant nitrogen gene was *narG*, which was mainly derived from uncultured bacteria, followed by *amoA*, which was largely derived from the bacterial phylum *Proteobacteria*, one of the largest divisions among prokaryotes^29^. *nifH* genes, which are involved in nitrogen fixation, were mostly derived from bacteria, except for some genes that were derived from the methanogenic *Euryarchaeota* phylum of the archaea, which is the only phylogenetic branch that fixes nitrogen^30^, and mainly includes *Methanothermobacter thermautotrophicus*, *Methanococcus maripaludis*, and *Methanosarcina barkeri*. The abundances of the *nir* gene, which is involved in assimilatory nitrogen reduction, the *narG* and *nirK* genes, which encode a copper-containing nitrite reductase, the *nosZ* gene, which is involved in denitrification, and the *gdh* gene, which is involved in ammonification, were significantly different between the CF and DBF, which indicates that these two forests have distinctly different mechanisms of nitrogen metabolism.

### Correlation networks of carbon and nitrogen cycling genes

We examined microbial carbon and nitrogen cycling genes via network analyses. For carbon cycling genes, although similar thresholds were used to construct the networks, the sizes of the CF, MBF, and DBF networks were different, containing 974, 1,187, and 1,334 carbon cycling genes, respectively (Supplementary Table S4). All curves of network connectivity obeyed the power-law model (R^2^ > 0.90). The top four functional genes with the highest connectivities in the three forest samples are shown in Supplementary Table S5. These include the carbon degradation genes *amyA*, *nplT-1* (neopullulanase), *nplT-2*, and exochitinase in the CF samples; the carbon degradation genes *amyA*, *ara-fungi*, phenol-oxidase, and mannanase in the MBF samples; and the carbon degradation genes pectinase and *amyA*, and the carbon fixation genes *RuBisCO* and *CODH* in the DBF samples (Supplementary Table S5, Supplementary Fig. S2A).

There were 743, 720, and 1,142 nitrogen cycling genes in the CF, MBF, and DBF networks, respectively (Supplementary Table S4). The nitrogen fixation gene *nifH*, the ammonification gene *ureC*, and the denitrification genes *nirS* and *nirK* were found in the CF, and the denitrification gene *nirK*, the dissimilatory nitrogen reduction gene *nrfA*, and the nitrogen fixation genes *nifH-1* and *nifH-2* were found in the MBF. The top four denitrification genes, *nirS*, *norB*, *nosZ*, and *narG*, were derived from four different species in the DBF (Supplementary Table S5, Supplementary Fig. S2B).

**Figure 3:**
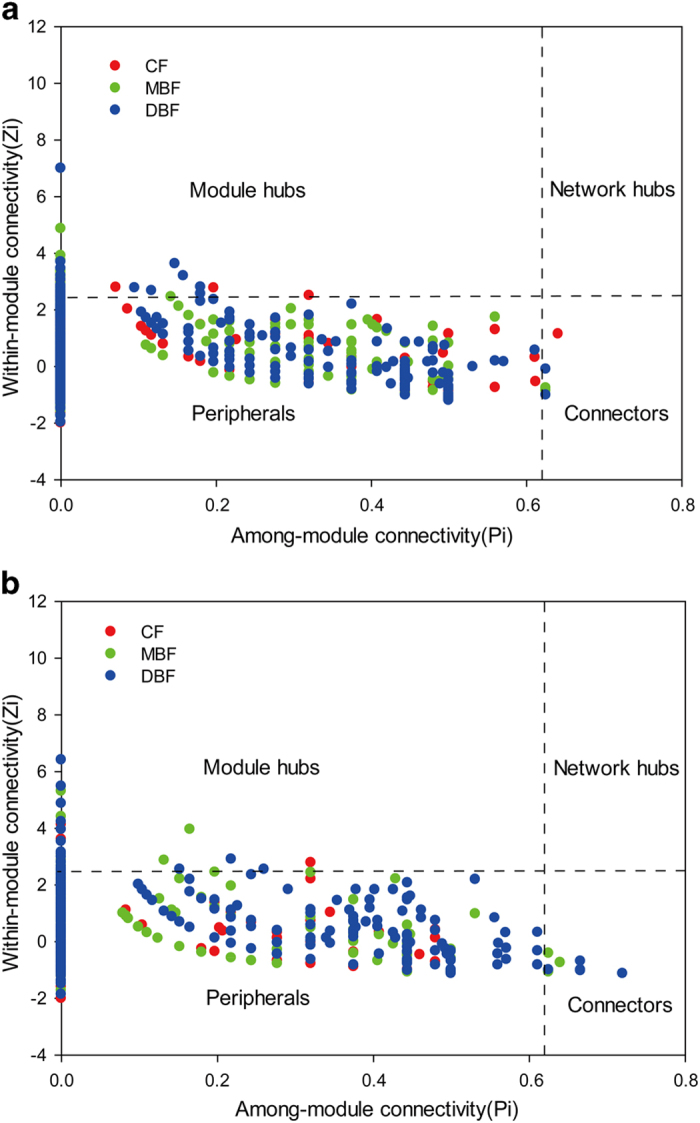
Z-P plot showing the distribution of functional genes involving in carbon (**a**) and nitrogen (**b**) cycling based on their topological roles. Each color represents a kind of forest type, respectively CF (red), MBF (green) and DBF (blue). The topological role of each gene was determined according to the scatter plot of within-module connectivity (Zi) and among-module connectivity (Pi).

Subsequently, the topological properties of the networks were examined (Fig. 3). Regarding the carbon cycling gene networks, the DBF had more hubs (11 genes) than the CF (9 genes) and MBF (9 genes). The DBF consistently had a higher number of nodes and links, and increased modularity (Supplementary Table S4). The findings were similar for the nitrogen cycling gene networks; there were 20, 11, and 17 hubs in the DBF, CF, and MBF, respectively.

### Relationship between the soil microbial community structure and environmental parameters

Mantel test analyses indicated that soil temperature, SOC, TN, and AN were significantly correlated with soil microbial communities at both the taxonomic and functional gene levels (*P* < 0.05) (Supplementary Table S1). Soil temperature was the most significantly correlated variable with soil microbial communities at the functional gene level (R = 0.294, *P* = 0.001). Plant diversity and soil moisture were significantly correlated with soil microbial communities at the taxonomic level (*P* < 0.05) (Supplementary Table S1). In addition, some phyla of the *Acidobacteria*, *Actinobacteria*, *Chlamydiae*, *Chloroflexi*, *Firmicutes*, *Planctomycetes*, *Proteobacteria*, and *Verrucomicrobia* were significantly and positively correlated with TE10 (Supplementary Table S6). Phyla such as the *Actinobacteria*, *Chloroflexi*, and *Proteobacteria* were significantly (*P* < 0.05) and positively correlated with SOC, TN, and AN (Supplementary Table S6).

**Figure 4:**
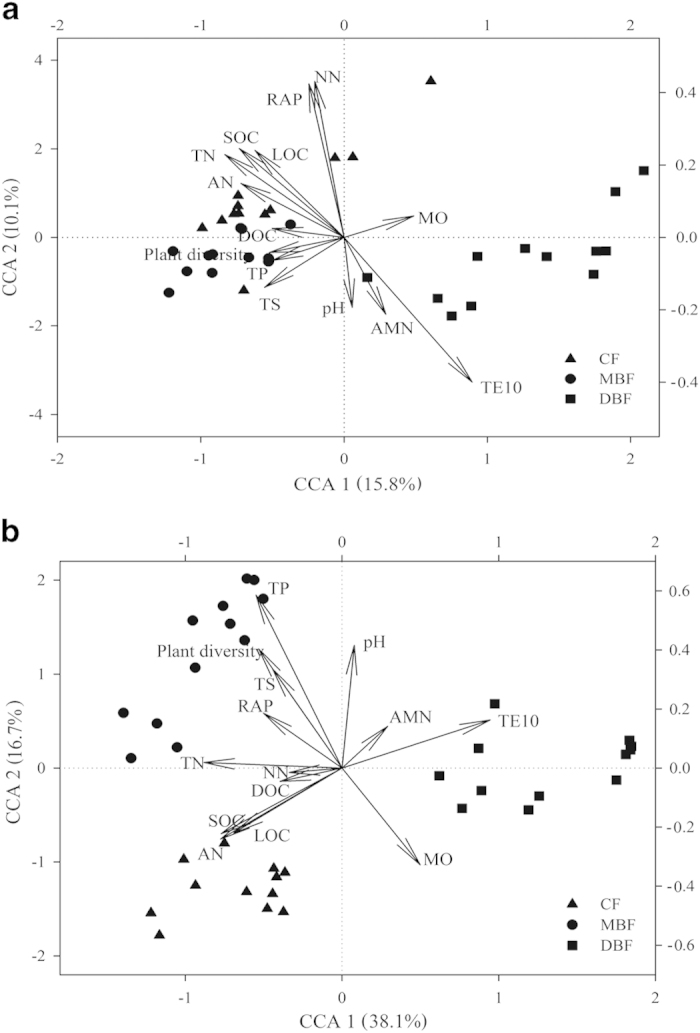
Canonical correspondence analysis (CCA) of (**a**) high-throughput sequencing data and (**b**) GeoChip data with environmental parameters. SOC - soil organic carbon, TN - total nitrogen, AN - available nitrogen, RAP - rapid available phosphorus, AMN - ammonium nitrogen, NN - nitrate nitrogen, MO - soil moisture, TE10 - soil temperature at the depth of 10 cm (° C), TS - total sulfur, TP - total phosphorus, DOC - dissolved organic carbon, LOC - labile organic carbon.

To further explore these relationships, we performed a canonical correlation analysis (CCA) based on the sequencing and GeoChip data (Fig. 4). The results of the CCA, which were significant at the confidence levels of *P* = 0.010 and *P* = 0.005 for the sequencing and GeoChip data, respectively, indicated that TE10, SOC, AN, and TN were significantly correlated with CCA axis 1 (*P* < 0.05) at both the taxonomic and functional gene levels based on their direction and magnitude, suggesting that they were important for explaining the variations between the microbial communities. Furthermore, TE10, followed by soil moisture, was the most important factor that influenced the soil microbial community of the DBF, as indicated by the direction and magnitude of the arrow.

## Discussion

Microbial communities in succession have been examined in different environments and on different timescales^2^,^13^,^14^. In general, microbial communities are able to respond more rapidly than plant communities to changing conditions during succession, and they are early signals of the recovery trajectory^12^. Given the high phylogenetic diversity, abundance, ubiquity, and biogeochemical importance of microbes, better integration of microbes into conceptual models of ecological succession is desired. Studying microbial communities helps us to understand how microbes play essential roles in biogeochemical cycling and ecosystem functioning^31^.

Microbial community structure and composition, which were closely linked to soil and vegetation properties (Fig. 4, Supplement Table S1), were distinctly different between the three forest types (Fig. 1, Supplement Table S3), thereby providing supporting evidence that distinct soil and vegetation properties could induce different soil microorganisms because of the formation of diverse microhabitats that support diverse collections of species^32^,^33^. Soil temperature could have an important effect on soil microbial community structure, as verified by the CCA model and Mantel test, at the functional and taxonomical levels (Fig. 4, Supplementary Table S1). Studies have documented that soil temperature could change the microbial community structure, such as the ratio of fungi to bacteria at a tallgrass prairie site^34^, and reduce the abundance of fungi, causing a shift toward Gram-positive bacteria and actinomycetes in a mineral soil microbial community after 15 years of soil warming^35^. Variations in soil temperature change the composition of microbial communities and influence heterotrophic respiration by changing the activity of extracellular enzymes, the microbial respiration rate, and the rate of microbial uptake of soluble substrates^32^,^36^. At higher temperatures, microbial populations have the ability to metabolize substrates that are not used by members of microbial communities at lower temperatures^32^. Soil moisture was another major factor that influences the microbial structure in the forest floor^32^, which was supported by our observation that soil moisture was significantly correlated with taxonomical composition (Supplementary Table S1). Moisture levels could influence soil microbial communities and control microbial activity across a range of environments, including saline water, food, wood, biofilms, and soils^36^,^37^. Soil moisture effectively enhances substrate fluxes to the cell surface, while low water availability can inhibit microbial activity by lowering intracellular water potential and enzymatic activities^37^. Soil temperature and moisture influenced soil respiration interdependently, and they also strongly controlled the rates of decomposition and nutrient mineralization^38^.

Plant species and their communities can also affect microbial community structures because of differences in canopy cover, rooting depth, and litter quality/quantity^33^,^39^. The decomposition of plant litter is an important process that is mediated by microbes. Variations in litter quality within and between plant species evoked differential microbial responses to quantitative (e.g., lignin concentrations) and qualitative (e.g., lignin composition) differences in plant compounds^40^. Notably, rates of leaf litter decomposition are regulated by a hierarchy of physical, chemical, and biotic factors that interact with fungi and bacteria, which are the main agents of decomposition^41^. Different vegetation types produce distinct quantities and qualities of plant litter, which mainly contributes to soil carbon and nitrogen. Our result found that soil microbial communities were significantly (*P* < 0.05) correlated with SOC, TN and AN (Supplementary Table S1), which was probably explained by decomposing the plant litter with different chemical structure. In general, the humus profile in deciduous and sclerophyllous forests is usually thinner than that in coniferous forests (such as the CF). Moreover, we found more genes and/or higher fungal diversity in the DBF than in the CF and MBF (Supplementary Table S7), which may have an advantage in utilizing nutrients from litter, which is consistent with the critical role of fungi in the degradation of recalcitrant litter carbon sources^42^. Therefore, plant species, especially plant litter, could have a close relationship with soil microbial communities.

Although correlation-based ecological network studies are still at the initial stage, they shed light on the cooperation or competition between microbial species, either directly or indirectly^43^. As described previously, such networks in microbial communities were defined as molecular ecological networks, in which different nodes (e.g., functional genes, OTUs) were linked by edges (interactions)^20^. Using GeoChip data, we investigated functional network interactions in microbial communities to assess their effects on three forest types, allowing us to evaluate microbial ecology within the context of different forest environments^44^. In network biology, a module is a group of species that interact strongly among themselves, but not with species in other modules^45^. Modularity in an ecological community may reflect habitat heterogeneity, physical contact, functional association, divergent selection, and/or the phylogenetic clustering of closely related species^45^. Our findings suggest that the DBF has a larger and tighter network (more modules and higher modularity) (Supplementary Table S4), implicating stronger coupling between microbes in mediating soil carbon and nitrogen cycles compared with the CF or MBF, which resulted in lower contents of soil nutrients, such as SOC or TN in the DBF (Supplementary Table S1). In addition, because “keystone” genes/species can be identified based on network topology and node memberships^46^, we proceeded to select the top four functional genes with high connectivities to construct carbon and nitrogen cycling networks. The carbon and nitrogen cycling networks in the DBF were more complicated than those in the CF and MBF. This could be because stronger microbial coupling could partially and indirectly be ascribed to soil temperature. Soil warming in the DBF resulted in relatively higher rates of microbial respiration in soil organic matter, suggesting that the DBF could more rapidly degrade soil organic matter in the presence of carbon inputs, thereby increasing the potential for carbon losses^47^. Meanwhile, the abundance and diversity of fungi, which are known to degrade recalcitrant organic materials from plant litter^48^, were significantly higher (*P* < 0.05) in the DBF than in the CF and MBF (Supplementary Table S7). This change could lead to reduced soil carbon storage, as shown in a previous study^49^. Therefore, the overall microbial community of the DBF suggests that it has a larger metabolic potential, higher sensitivity in response to environmental changes, and greater robustness when random and specific perturbations affect its functional stability. Metabolic network analysis is an emergent, evolving procedure for data-mining a large amount of macro data. Although it has been interpreted to reveal microbial interactions^19^, it remains a conceptual framework that needs experimental evidence, which is difficult to generate using current technologies. Thus, the use of networks serves to provide insights regarding keystone genes or network topological structures that cannot be identified otherwise.

In conclusion, we report the profiling of soil microbial communities in a DBF, MBF, and CF, which are the most widely distributed forests in the world, and which might be used as indicators of environmental changes. We found that temperature was important in shaping microbial communities at both the taxonomic and functional gene levels. This study represents a step forward in our understanding of microbial communities, and it provides valuable insights regarding the potential effects of environmental changes and vegetation succession on primary forest ecosystems.

## Methods

### Site description and sampling

Samples were taken from three adjacent forest types, including a CF, a MBF, and a DBF, in the Shennongjia National Nature Reserve (SNNR, 110°17′–110°21′ E, 31°28′–31°29′ N) on Shennongjia Mountain, Hubei province, central China, in August 2011). The mean annual temperature and mean annual precipitation of the sampling area were obtained from the WorldClim-Global Climate Data ( http://www.worldclim.org/). All spatial geographical coordinates and altitudes were recorded by GPS (eTrex Venture, Garmin, Lenexa, KS, USA). These sampling sites were selected for their representative vegetation and soil attributes. Detailed site information is listed in Table 1. At each site, 12 plots (20 m × 20 m) were established in an approximate area covering 3 ha^2^, with a distance of approximately 20 m between adjacent plots, and we collected a total of 36 soil samples at a 0–10-cm depth during the peak plant growth stage. Soil samples were sieved using a 2-mm mesh to remove roots and stones, homogenized, and stored at 4 °C for soil physicochemical measurements and at −80 °C for DNA extraction.

### Soil and vegetation measurements

Soil properties were measured as previously described^50^, including dissolved organic carbon (DOC), LOC, AN, ammonium nitrogen (AMN), NN, total phosphorus (TP), RAP, and total sulfur (TS). SOC and TN were measured using the wet oxidation and the modified Kjeldahl procedure, respectively, as previous described^51^. Soil pH was measured at a water-to-soil mass ratio of 2.5:1 using a pH meter with a calibrated combined glass electrode^52^. Soil moisture (MO) was determined gravimetrically by weighing, after drying in an oven at 105 °C for 10 h. Soil temperature (TE10) was measured by a Hobo Temperature instrument at a depth of 10 cm. Plant properties in each plot were surveyed, including plant species, tree number and height, diameter at breast height (DBH), or ground diameter. Plant species diversity was characterized using Shannon’s and Simpson’s indices. All these variables were averaged to obtain site-level estimates using the mean values of each sample.

### DNA extraction, purification, and quantitation

Soil DNA was extracted from each of the 0.5-g soil samples using the MO BIO PowerSoil^®^ DNA isolation kit (MO BIO Laboratories, Carlsbad, CA, USA) according to the manufacturer’s protocol. Soil DNA was extracted by freeze-grinding mechanical lysis as described previously^53^. The freshly extracted DNA was purified twice using 0.5% low melting point agarose gels, followed by phenol-chloroform-butanol extraction. DNA quality was assessed using 260 nm/280 nm and 260 nm/230 nm ratios, and final DNA concentrations were quantified by the PicoGreen method using a FLUOstar Optima microplate reader (BMG Labtech, Jena, Germany).

### GeoChip hybridization and data processing

Purified DNA was labeled with Cy3, dried, rehydrated, and hybridized with GeoChip 4.0 as described previously^54^. These arrays were scanned with 100% laser power and a photomultiplier tube using a MS 200 Microarray Scanner (Roche, Basel, Switzerland). The signal intensity of each spot was quantified using ImaGene software (Arrayit, Sunnyvale, CA, USA), and poor-quality spots with a signal/noise ratio (SNR) < 2.0 were discarded prior to statistical analyses^54^.

Raw GeoChip data were uploaded to the GeoChip data analysis manager (http://ieg.ou.edu/microarray/) and pre-processed using the data analysis pipeline as previously described^54^. The following major steps were performed: (i) removal of poor quality spots (SNR < 2.0); (ii) normalization of the signal intensity of each spot based on the mean signal intensity across all genes on the arrays; and (iii) transformation of the data using the natural logarithmic form. Pre-processed GeoChip data were used for further statistical analysis.

### Illumina sequencing and data processing

Sequencing of PCR amplicons of 16S rRNA was conducted with the Illumina MiSeq platform (Illumina, San Diego, CA, USA) targeting the V4 hyper variable regions (forward primer, 515F, 5′-GTGCCAGCMGCCGCGGTAA-3′; reverse primer, 806R, 5′-GGACTACHVGGGTWTCTAAT-3′) for 2 × 150-bp paired-end sequencing (Illumina)^55^,^56^. PCR amplification was performed in triplicate in 25-μl reactions under the following conditions: initial denaturation at 94 °C for 1 min, followed by 30 cycles of 94 °C for 20 s, 53 °C for 25 s, 68 °C for 45 s, and a final extension step of 10 min at 68 °C. The PCR amplicons from all samples were pooled in equimolar ratios. Primers and primer dimers were separated from the combined PCR products by electrophoresis on 1% agarose gels, and PCR products were purified using the QIAquick gel extraction kit (Qiagen, Valencia, CA, USA). More details regarding the PCR amplifications were provided by Ding^57^.

Raw sequences were divided into samples by barcodes (up to one mismatch was permitted) using the Galaxy Illumina sequencing pipeline ( http://rccc.ou.edu). Quality trimming was performed using Btrim^58^. Forward and reverse reads were incorporated into full-length sequences with FLASH^59^. Sequences that were too short or that contained ambiguous bases were removed. OTUs were classified by UCLUST at the 97% similarity level^60^, and singletons were removed. Rarefaction analysis was conducted using the original detected OTUs. The taxonomic assignment was performed by the RDP classifier^61^ with minimal 50% confidence estimates. Random resampling was conducted on 20,000 sequences per sample.

### Statistical analysis

All statistical tests were performed in R v. 3.1.2 using the Vegan package (v.2.2-1). The extracted climate data were analysed using the spatial analysis tool of ArcGIS version 9.3 (ESRI, Redlands, CA, USA). The richness of total soil microbial communities was calculated by counting all samples, including only one being in any forest sites, while the richness in one forest type calculated by counting each plot of each forest type, then by averaging the 12 plots of each forest type. Shannon’s and Simpson’s indices were used to evaluate plant and soil microbial community diversity. DCA was used to estimate microbial community composition heterogeneity. Bray–Curtis distances were used to test for dissimilarities between any two of the three forest types by ANOSIM. Mantel tests and CCA were used to evaluate the linkages between soil microbial structure and environmental attributes. To select attributes for the CCA model, we used variation inflation factors (VIFs) to examine whether the variance of the canonical coefficients was inflated by the presence of correlations with environmental variables. The VIFs were used to remove redundant parameters stepwise in the CCA modeling^17^.

As previously described^21^, random matrix theory-based approaches were used for network construction, hub and connector gene identification, and topological property determination with an automatic threshold. To ensure correlation reliability, probes detected in at least 9 out of the 12 replicates were used for network analysis, and network graphs were visualized by Cytoscape 2.6.0 software^62^. Various network properties, such as average degree, average path distance, average clustering coefficient, and modularity index, were calculated^20^. Modularity was described to be a network that could be naturally divided into communities or modules^45^. Each module in the gene networks is supposed to be a functional unit that contains several elementary genes and that executes an identifiable task^20^. The network modules were generated using rapid greedy modularity optimization^45^. Hub and connector genes were determined by among-module connectivity (Pi) and within-module connectivity (Zi). Module hubs were defined as nodes with Pi ≤ 0.62 and Zi > 2.5, with connectors defined as nodes with Pi > 0.62 and Zi ≤ 2.5, and network hubs defined as nodes with Pi > 0.62 and Zi > 2.5. Peripheral nodes were defined as nodes with Pi ≤ 0.62 and Zi ≤ 2.5.

## Author Contributions

J.C. carried out the data analysis and wrote the article. Y.Z., Y.Y. and J.Z. developed the original ideas, contributed part of the discussion and oversight the work.J.C., H. L., X. L., D. L. and Y. Z. contributed sample collection and processing.X. L. and H. Y. the readand approved the final version.

## Additional Information

**How to cite this article**: Cong, J. *et al.* Analyses of soil microbial community compositions and functional genes reveal potential consequences of natural forest succession. *Sci. Rep.* **5**, 10007; doi: 10.1038/srep10007 (2015).

## Supplementary Material

Supporting Information Supplementary Figures and Supplementary Tables S1-S7

## Figures and Tables

**Table 1:** Sites information in this study.

**Study site**	**Vegetation type**	**Dominant community of tree and shrubs**	**Elevation (m)**
Jinhouling	Coniferous forest (CF)	*Abies fargesii-Syringa reflexa*	2545 ± 55
Shennongyuan	Mixed broadleaf forest (MBF)	*Acer maximowiczii-Schisandra incarnata*	2295 ± 31
Jiuhuping	Deciduous Broadleaved forest (DBF)	*Carpinus viminea-Viburnum erosum*	1784 ± 35

**Table 2:** Summary of number and diversity index of soil microbial communities based on GeoChip 4.0 and 16S sequencing data.

**Forest types**		**GeoChip 4.0**		**16S OTUs**	
**Richness**^c^	**Shannon Index**	**Richness**^c^	**Shannon Index**		
CF	34221 ± 3916b^d^	10.43 ± 0.12b	6542 ± 933ab	7.10 ± 0.60b
MBF	34153 ± 3551b	10.43 ± 0.11b	6210 ± 729b	6.72 ± 0.60b
DBF	39970 ± 2250a	10.59 ± 0.06a	7150 ± 945a	7.61 ± 0.42a

Data present the mean value and standard error.

^c^Detected gene/sequence numbers.

^d^Significant differences among forest types are indicated by alphabetic letters, *P* < 0.05.
