# Supplementary material for: Analyses of soil microbial community compositions and functional genes reveal potential consequences of natural forest succession
Source: Sci Rep. 2015 May 6;5:10007. doi: 10.1038/srep10007 (PMC4421864; doi:10.1038/srep10007)
Supplement: Supporting Information — Supplementary Figures and Supplementary Tables S1-S7 [file srep10007-s1.doc]

**Analyses of soil microbial community compositions and functional genes reveal potential consequences of natural forest succession**

Jing Cong^1,2^, Yunfeng Yang^3^, Xueduan Liu^2^, Hui Lu^1^, Xiao Liu^1^, Jizhong Zhou^3,4^, Diqiang Li^1^, Huaqun Yin^2^, Junjun Ding^3^ & Yuguang Zhang^1^*

^1^Institute of Forestry Ecology, Environment and Protection, and the Key Laboratory of Forest Ecology and Environment of State Forestry Administration, the Chinese Academy of Forestry, Beijing 100091, China

^2^School of Minerals Processing and Bioengineering, Central South University, Changsha 410083

^3^State Key Joint Laboratory of Environment Simulation and Pollution Control, School of Environment, Tsinghua University, Beijing 100084, China

^4^Institute for Environmental Genomics and Department of Botany and Microbiology, the University of Oklahoma, Norman OK 73019

*To whom correspondence may be addressed. E-mail: yugzhang@sina.com.cn;

Phone: +86-010-62889240; Fax: +86-010-62884972

Subject Category: Microbial ecology and functional diversity of natural forest

Type: research article


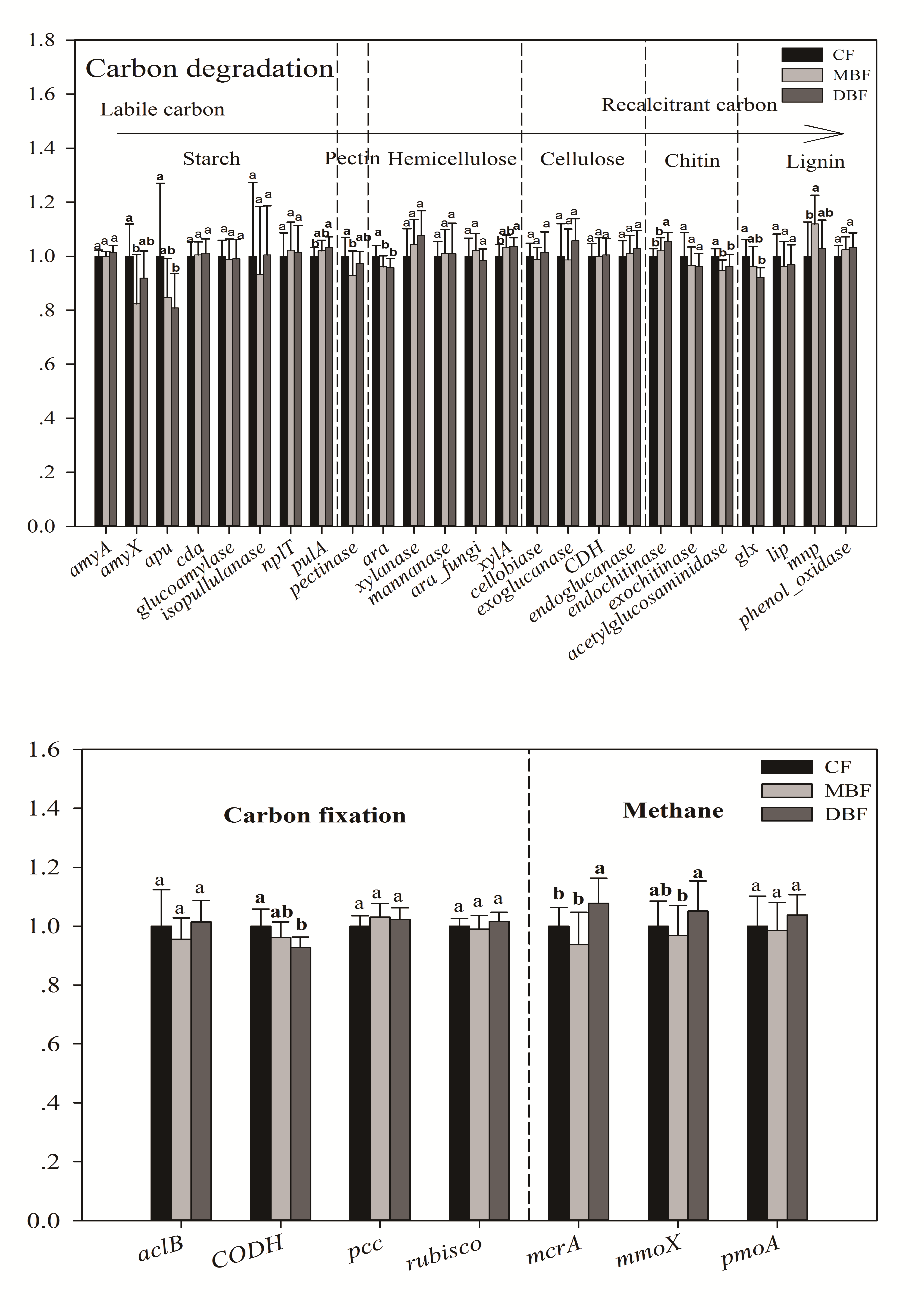


Supplementary Figure S1 The relative abundance of detected genes involved in carbon cycling of three adjacent natural forest types. All data are presented as the mean value and standard error. Significant differences among forest types are indicated by alphabetic letters.


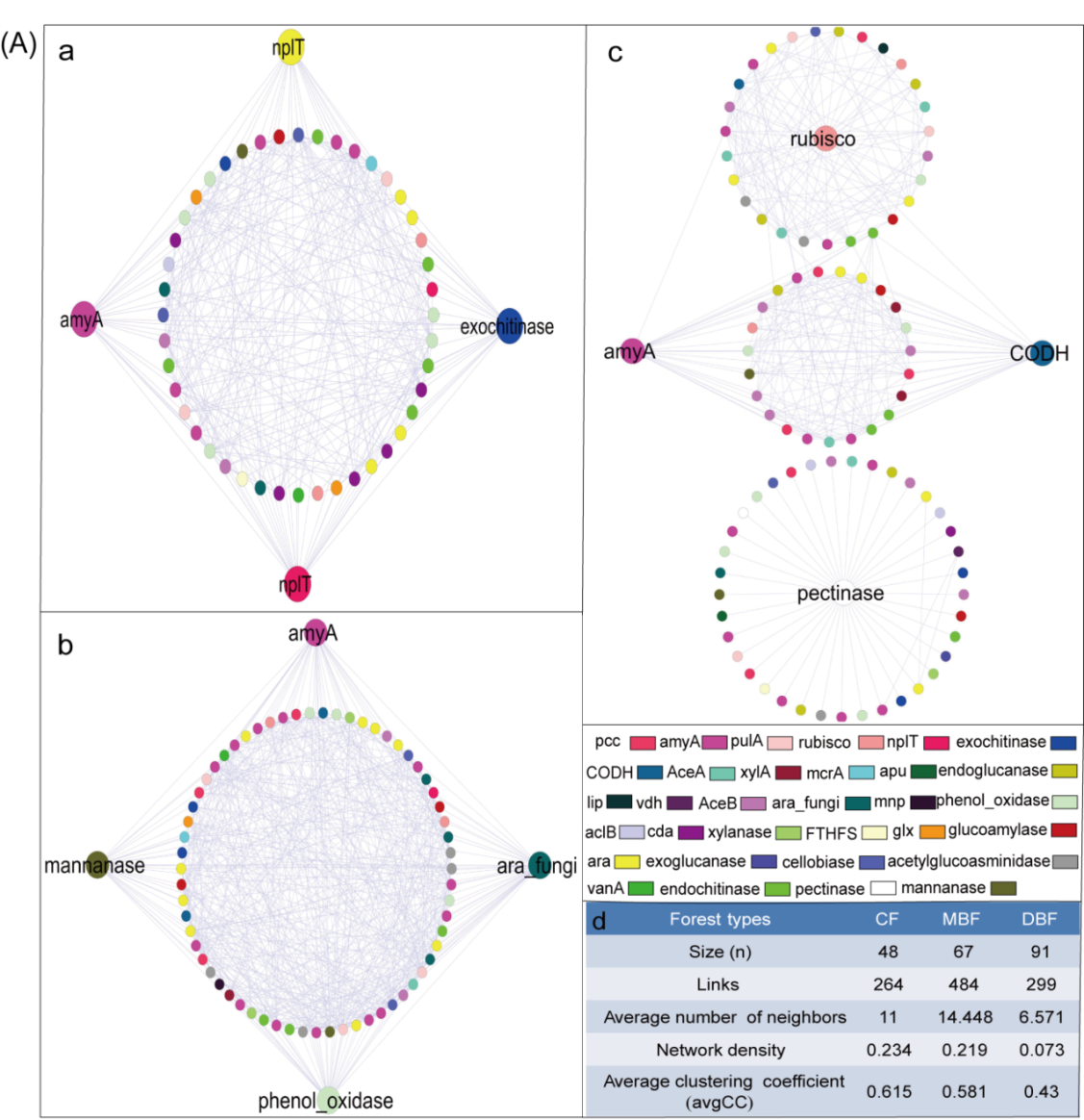


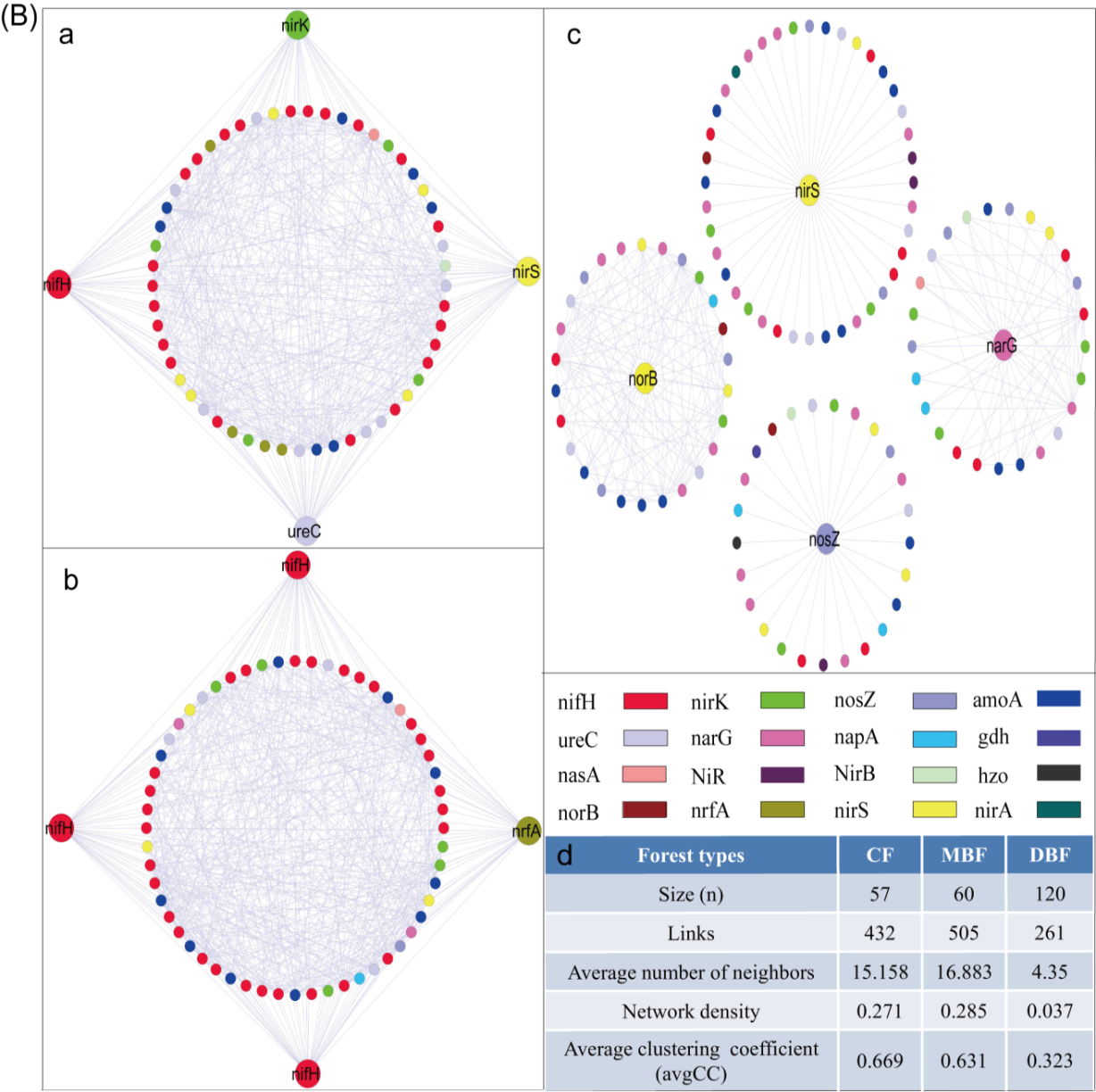


Supplementary Figure S2 Network interactions involving in top four functional genes with the highest connectives of carbon (A) and nitrogen (B) cycling gene categories, respectively in (a) CF, (b) MBF and (c) DBF based on RMT-based approach with the GeoChip data. Each node expresses a functional gene and different colors present diverse functional genes. These genes were divided into different modules as highly connected functional units by using the greedy modularity optimization method. (d) The topological properties of gene networks in comparison.

Supplementary Table S1 Diversity index of plant communities and soil environmental factors analysed by ANOVA and Mantel tests of GeoChip and 16S sequencing data with environmental properties among three adjacent natural forest types.

| Environmental variable | | Forest types | | | R/*P* value | |
| --- | --- | --- | --- | --- | --- | --- |
|  |  | CF | MBF | DBF | GeoChip 4.0 | 16S OTUs |
| Plant (vascular plants) | Shannon diversity | 1.76±0.72b | 2.45±0.35a | 1.95±0.73b | 0.127/0.077 | **0.280/0.001** |
|  | Simpson diversity | 4.31±2.03b | 7.53±3.28a | 2.59±3.40b | 0.062/0.198 | **0.459/0.001** |
| Soil physical and chemical factors | SOC^c^ (g/kg) | 61.58±16.74a^d^ | 53.06±9.62a | 30.53±3.58b | **0.270/0.002** | **0.104/0.046** |
|  | TN(g/kg) | 4.22±0.85a | 4.21±0.62a | 1.94±0.32b | **0.258/0.001** | **0.297/0.001** |
|  | AN(mg/kg) | 340.85±79.80a | 305.97±55.64a | 192.06±37.09b | **0.236/0.005** | **0.127/0.021** |
|  | RAP(mg/kg) | 10.81±14.64a | 13.73±7.23a | 3.21±0.36b | 0.199/0.067 | 0.110/0.056 |
|  | NN(mg/kg) | 17.68±14.44a | 12.06±13.99a | 5.14±6.24b | 0.086/0.753 | 0.059/0.166 |
|  | AMN(mg/kg) | 15.10±3.32b | 17.66±5.20ab | 19.02±3.87a | **0.153/0.043** | 0.030/0.680 |
|  | pH | 5.07±0.48a | 5.36±0.31a | 5.27±0.47a | 0.116/0.112 | 0.065/0.138 |
|  | MO (%) | 44.42±4.23a | 40.23±6.55b | 48.95±4.56a | 0.064/0.741 | **0.126/0.041** |
|  | TE10 (℃) | 10.83±0.50c | 11.74±0.80b | 16.30±0.44a | **0.294/0.001** | **0.232/0.002** |
|  | TS(mg/kg) | 333.40±72.84b | 476.58±189.98a | 227.81±188.86b | 0.053/0.273 | **0.125/0.032** |
|  | TP(mg/kg) | 600.64±109.59b | 1469.98±943.87a | 241.92±42.77b | 0.081/0.210 | **0.204/0.006** |
|  | DOC(mg/kg) | 206.23±93.19a | 209.61±45.80a | 150.61±47.93a | 0.066/0.749 | 0.089/0.956 |
|  | LOC(g/kg) | 6.44±3.42a | 4.59±1.75a | 1.48±6.33b | **0.267/0.017** | 0.060/0.148 |

Data present the mean value and standard error.

^c^Abbreviations: CF - coniferous forest, MBF - mixed broadleaf forest and DBF - deciduous broadleaf forest, SOC - soil organic carbon, TN - total nitrogen, AN - available nitrogen, RAP - rapid available phosphorus, AMN - ammonium nitrogen, NN - nitrate nitrogen, MO - soil moisture, TE10 - soil temperature at a depth of 10cm (°C), TS - total sulfur, TP - total phosphorus, DOC - dissolved organic carbon, LOC - labile organic carbon.

^d^Significant differences among forest types are indicated by alphabetic letters, *P* < 0.05.

Supplementary Table S2 Number of OTU detected by illumina sequencing of 16S rRNA tags in major phyla

| Domain and phylum | Total (%) | Number of OTUs | | |
| --- | --- | --- | --- | --- |
|  |  | CF | MBF | DBF |
| *Acidobacteria* | 10899 (14.73) | 1177.58a^c^±211.29 | 947.67b±188.70 | 1132.25a±178.15 |
| *Actinobacteria* | 5948（8.04） | 487.00b±175.90 | 435.58b±99.39 | 663.92a±143.60 |
| *Armatimonadetes* | 242（0.33） | 15.08a±4.83 | 16.42a±8.33 | 18.33a±6.81 |
| *Bacteroidetes* | 2830（3.82） | 264.58a±100.31 | 293.50a±73.86 | 250.17a±102.66 |
| *Chlamydiae* | 528 (0.71) | 27.67a±10.11 | 16.83b±5.62 | 26.67a±8.24 |
| *Chloroflexi* | 1073 (1.45) | 99.58a±18.05 | 82.75a±31.89 | 60.25b±16.01 |
| *Crenarchaeota* | 135 (0.18) | 24.83a±10.46 | 16.00b±12.74 | 8.67b±5.02 |
| *Firmicutes* | 1205 (1.63) | 100.08a±20.54 | 103.00a±17.92 | 108.75a±22.88 |
| *Gemmatimonadetes* | 663 (0.90) | 69.50a±22.84 | 60.58ab±20.55 | 51.50b±15.90 |
| *Planctomycetes* | 5185 (7.01) | 306.58b±108.73 | 275.83b±58.40 | 577.92a±115.49 |
| *Proteobacteria* | 32016 (43.27) | 2828.17a±303.75 | 2901.83a±292.92 | 2944.17a±450.07 |
| *Verrucomicrobia* | 5062 (6.84) | 529.50b±109.66 | 542.67b±124.78 | 739.75a±151.93 |
| Unclassified | 7796(10.54) | 578.25a±119.66 | 485.25b±90.96 | 544.83ab±77.87 |
| Others | 411 (0.56) | 20.25a±8.08 | 19.92a±7.20 | 14.67b±3.75 |
| Total | 73993 | 6541.75ab±933.38 | 6209.92b±728.88 | 7149.50a±944.82 |

^c^denotes that significant differences are indicated by alphabetic letters in the phylotypes or all detected OTUs among forest types, *P* < 0.05. Data present the mean value and standard error.

Supplementary Table S3 Statistical analysis of differences in the microbial community composition and structure among three adjacent natural forest types.

| Forest communities | GeoChip 4.0 | | 16S OTUs | |
| --- | --- | --- | --- | --- |
|  | MRPP.sig | ANOSIM.sig | MRPP.sig | ANOSIM.sig |
| CF vs. MBF | 0.024 | 0.04 | 0.003 | 0.001 |
| CF vs. DBF | 0.015 | 0.006 | 0.000999 | 0.003 |
| MBF vs. DBF | 0.000999 | 0.001 | 0.000999 | 0.001 |

Supplementary Table S4 Topological properties of carbon and nitrogen cycling gene networks

| Network indexes | Carbon cycling | | | Nitrogen cycling | | |
| --- | --- | --- | --- | --- | --- | --- |
|  | CF | MBF | DBF | CF | MBF | DBF |
| Threshold | 0.940 | 0.940 | 0.951 | 0.933 | 0.939 | 0.939 |
| Modularity | 0.852 | 0.778 | 0.819 | 0.772 | 0.708 | 0.789 |
| Total nodes | 974 | 1187 | 1334 | 743 | 720 | 1142 |
| Total links | 1432 | 2234 | 2460 | 1315 | 2234 | 2460 |
| R square of power-law | 0.949 | 0.910 | 0.925 | 0.918 | 0.910 | 0.925 |
| Average degree | 2.940 | 3.764 | 3.688 | 3.540 | 3.764 | 3.688 |
| Average clustering coefficient | 0.194 | 0.220 | 0.229 | 0.184 | 0.220 | 0.229 |
| Average path distance | 2.370 | 5.080 | 3.210 | 1.365 | 5.080 | 3.210 |
| Geodesic efficiency | 0.034 | 0.064 | 0.062 | 0.038 | 0.064 | 0.062 |
| Harmonic geodesic distance | 29.064 | 15.527 | 16.189 | 26.620 | 15.527 | 16.189 |
| Density | 0.003 | 0.003 | 0.003 | 0.005 | 0.003 | 0.003 |
| Transitivity | 0.375 | 0.370 | 0.358 | 0.454 | 0.370 | 0.358 |

Supplementary Table S5 The top four functional genes with highest connectivity involving in the carbon and nitrogen cycling in three forest types

| Forest types | Carbon cycling | | | Nitrogen cycling | | |
| --- | --- | --- | --- | --- | --- | --- |
|  | Gene | Sub-category | Organism | Gene | Sub-category | Organism |
| CF | *nplT*(1) | Carbon degradation | *Streptomyces* sp. Mg1 | *nifH* | Nitrogen fixation | *Clostridium* sp. MK31 |
|  | *amyA* |  | *Thermoanaerobacter* sp. X514 | *ureC* | Ammonification | *Arthrospira maxima* CS-328 |
|  | *nplT*(2) |  | *Geobacillus* sp. Y412MC10 | *nirS* | Denitrification | uncultured bacterium |
|  | *exochitinase* |  | *Oribacterium sinus* F0268 | *nirK* |  |  |
| MBF | *amyA* | Carbon degradation | *Erythrobacter litoralis* HTCC2594 | *nirK* | Denitrification | uncultured bacterium |
|  | *ara-fungi* |  | *Aspergillus clavatus* NRRL 1 | *nrfA* | Dissimilatory N reduction |  |
|  | *phenol-*  *oxidase* |  | uncultured fungus | *nifH*(1) | Nitrogen fixation |  |
|  | *mannanase* |  | *Bacteroides fragilis* 3_1_12 | *nifH*(2) |  |  |
| DBF | *pectinase* | Carbon degradation | *Colletotrichum gloeosporioides* f. sp. *Malvae* | *nirS* | Denitrification | uncultured bacterium |
|  | *amyA* |  | *Nocardioides* sp. JS614 | *norB* |  | *Chromobacterium violaceum* ATCC 12472 |
|  | *rubisco* | Carbon fixation | *Burkholderia phymatum* STM815 | *nosZ* |  | *Anaeromyxobacter dehalogenans* 2CP-C |
|  | CODH |  | *Ralstonia metallidurans* CH34 | *narG* |  | unidentified bacterium |

Supplementary Table S6 The correlation between phyla and environmental factors by Mantel tests among three adjacent natural forest types.

| Domain and phylum | TE10 (r,*P*) | MO (r,*P*) | pH (r,*P*) | SOC  (r,*P*) | TN  (r,*P*) | AN  (r,*P*) | AMN  (r,*P*) | LOC (r,*P*) | DOC (r,*P*) | Plant diversity (r,*P*) |
| --- | --- | --- | --- | --- | --- | --- | --- | --- | --- | --- |
| *Acidobacteria* | **0.256,0.002** | -0.045,0.605 | 0.203,0.082 | 0.146,0.077 | **0.196,0.010** | 0.051,0.259 | 0.014,0.384 | **0.232,0.040** | -0.086,0.783 | 0.127,  0.138 |
| *Actinobacteria* | **0.237,0.001** | 0.007,0.404 | **0.480,0.001** | **0.151,0.045** | **0.231,0.002** | **0.147,0.041** | -0.027,0.557 | 0.166,0.071 | -0.006,0.463 | 0.118,  0.124 |
| *Armatimonadetes* | 0.079,0.129 | -0.096,0.771 | 0.181,0.102 | -0.022,0.586 | 0.006,0.473 | -0.056,0.700 | -0.031,0.582 | -0.005,0.466 | **0.213,0.047** | 0.092,  0.211 |
| *Bacteroidetes* | 0.062,0.195 | -0.176,0.987 | **0.456,0.002** | **0.209,0.038** | -0.004,0.508 | 0.022,0.388 | **0.275,0.022** | **0.398,0.006** | 0.172,0.087 | -0.157,  0.958 |
| *Chlamydiae* | **0.143,0.023** | -0.011,0.500 | -0.048,0.630 | -0.007,0.494 | 0.034,0.318 | 0.035,0.342 | -0.192,0.963 | -0.013,0.512 | 0.172,0.082 | **0.315,**  **0.004** |
| *Chloroflexi* | **0.170,0.022** | -0.107,0.896 | -0.015,0.465 | **0.178,0.048** | **0.163,0.016** | **0.166,0.037** | 0.042,0.294 | 0.136,0.095 | 0.202,0.067 | -0.022,  0.497 |
| *Crenarchaeota* | -0.043,0.667 | -0.165,0.996 | -0.061,0.653 | 0.094,0.163 | -0.013,0.520 | **0.168,0.041** | -0.017,0.449 | -0.028,0.465 | 0.149,0.102 | **-0.131,**  **0.935,** |
| *Firmicutes* | **0.432,0.001** | -0.002,0.412 | -0.026,0.537 | 0.149,0.053 | **0.299,0.001** | **0.257,0.002** | -0.076,0.771 | 0.062,0.215 | 0.014,0.373 | **0.209,**  **0.037** |
| *Gemmatimonadetes* | 0.005,0.421 | -0.155,0.985 | -0.009,0.413 | 0.019,0.335 | -0.032,0.629 | 0.005,0.417 | 0.144,0.117 | -0.006,0.417 | 0.230,0.059 | -0.137,  0.943 |
| *Planctomycetes* | **0.473,0.001** | 0.051,0.254 | 0.025,0.361 | 0.019,0.354 | **0.352,0.001** | 0.133,0.070 | -0.074,0.735 | 0.072,0.209 | -0.101,0.825 | **0.229,**  **0.029** |
| *Proteobacteria* | **0.219,0.001** | **0.126,0.025** | 0.056,0.171 | **0.099,0.045** | **0.290,0.002** | **0.122,0.022** | -0.029,0.683 | 0.053,0.168 | -0.086,0.955 | 0.069,  0.110 |
| *Verrucomicrobia* | **0.402,0.001** | 0.125,0.116 | 0.041,0.272 | 0.135,0.071 | **0.191,0.016** | **0.191,0.010** | -0.050,0.674 | 0.099,0.147 | -0.160,0.979 | 0.124,  0.108 |
| Unclassified | **0.325,0.001** | -0.048,0.641 | **0.265,0.027** | **0.273,0.008** | **0.309,0.001** | **0.227,0.006** | 0.080,0.203 | **0.299,0.024** | 0.033,0.327 | -0.017,  0.511 |
| Others | 0.003,0.436 | -0.055,0.659 | **0.382,0.018** | **0.304,0.018** | 0.030,0.340 | 0.092,0.153 | 0.199,0.056 | **0.463,0.013** | 0.163,0.101 | -0.091,  0.789 |

Supplementary Table S7 Summary of number and diversity index of soil fungi based on GeoChip 4.0 data

| Soil fungi | CF | MBF | DBF |
| --- | --- | --- | --- |
| Richness | 26952.08±3413.46b^c^ | 27160.07±3678.69b | 32807.16±2173.69a |
| Diversity  (Shannon index) | 7.96±0.15b | 7.97±0.15b | 8.19±0.07a |
| Diversity  (Simpson index) | 2880.55±402.61b | 2911.66±427.53b | 3583.55±255.15a |

^c^Significant differences indicated by alphabetic letters, *P* < 0.05. Data present the mean value and standard error.
